# Loss of *STK11* Suppresses Lipid Metabolism and Attenuates *KRAS*-Induced Immunogenicity in Patients with Non–Small Cell Lung Cancer

**DOI:** 10.1158/2767-9764.CRC-24-0153

**Published:** 2024-08-30

**Authors:** Daniel R. Principe, Mary M. Pasquinelli, Ryan H. Nguyen, Hidayatullah G. Munshi, Alicia Hulbert, Alexandre F. Aissa, Frank Weinberg

**Affiliations:** 1 Department of Medicine, University of Wisconsin, Madison, Wisconsin.; 2 Division of Pulmonary, Critical Care, Sleep and Allergy, University of Illinois at Chicago, Chicago, Illinois.; 3 Division of Hematology and Oncology, University of Illinois Chicago and Translational Oncology Program, University of Illinois Cancer Center, Chicago, Illinois.; 4 Department of Medicine, Feinberg School of Medicine, Northwestern University, Chicago, Illinois.; 5 The Robert H. Lurie Comprehensive Cancer Center, Chicago, Illinois.; 6 Jesse Brown VA Medical Center, Chicago, Illinois.; 7 Department of Surgery, University of Illinois Chicago, Chicago, Illinois.; 8 Division of Genetics, Department of Morphology and Genetics, Federal University of São Paulo, São Paulo, Brazil.

## Abstract

As many as 30% of the patients with non–small cell lung cancer harbor oncogenic *KRAS* mutations, which leads to extensive remodeling of the tumor immune microenvironment. Although co-mutations in several genes have prognostic relevance in *KRAS*-mutated patients, their effect on tumor immunogenicity are poorly understood. In the present study, a total of 189 patients with non–small cell lung cancer underwent a standardized analysis including IHC, whole-exome DNA sequencing, and whole-transcriptome RNA sequencing. Patients with activating *KRAS* mutations demonstrated a significant increase in PDL1 expression and CD8^+^ T-cell infiltration. Both were increased in the presence of a co-occurring *TP53* mutation and lost with *STK11* co-mutation. Subsequent genomic analysis demonstrated that *KRAS/TP53* co-mutated tumors had a significant decrease in the expression of glycolysis-associated genes and an increase in several genes involved in lipid metabolism, notably *lipoprotein lipase*, *low*-*density lipoprotein receptor*, and *LDLRAD4*. Conversely, in the immune-excluded *KRAS/STK11* co-mutated group, we observed diminished lipid metabolism and no change in anaerobic glycolysis. Interestingly, in patients with low expression of *lipoprotein lipase*, *low*-*density lipoprotein receptor*, or *LDLRAD4*, *KRAS* mutations had no effect on tumor immunogenicity. However, in patients with robust expression of these genes, *KRAS* mutations were associated with increased immunogenicity and associated with improved overall survival. Our data further suggest that the loss of *STK11* may function as a metabolic switch, suppressing lipid metabolism in favor of glycolysis, thereby negating *KRAS*-induced immunogenicity. Hence, this concept warrants continued exploration, both as a predictive biomarker and potential target for therapy in patients receiving ICI-based immunotherapy.

**Significance:**

In patients with lung cancer, we demonstrate that *KRAS* mutations increase tumor immunogenicity; however, *KRAS/STK11* co-mutated patients display an immune-excluded phenotype. *KRAS/STK11* co-mutated patients also demonstrated significant downregulation of several key lipid metabolism genes, many of which were associated with increased immunogenicity and improved overall survival in *KRAS*-mutated patients. Hence, alteration to lipid metabolism warrants further study as a potential biomarker and target for therapy in patients with *KRAS*-mutated lung cancer.

## Introduction

Non–small cell lung cancer (NSCLC) is the leading cause of cancer-related death in the United States (1). Although NSCLC is genetically heterogeneous, as many as 30% of patients with NSCLC harbor gain-of-function mutations in the *KRAS* oncogene (2). Recent data demonstrate that gain-of-function *KRAS* mutations lead to extensive remodeling of the tumor immune microenvironment in several tumor types (3). However, in NSCLC, the effects of *KRAS* mutations on tumor immunity are multifaceted and often contradictory (4, 5). Although some studies seem to support an immunosuppressive role for KRAS in NSCLC tumors (6), there is a growing body of clinical evidence suggesting that *KRAS*-mutated tumors may be associated with favorable responses to immune checkpoint inhibitors (ICI; ref. 2) For example, recent clinical studies have shown that *KRAS*-mutated lung cancers treated with ICI-based immunotherapy demonstrated objective response rates of up to 41%, which was associated with 1-year progression-free survival rates of 12% to 28% (7, 8). However, other studies have found that the comparative efficacy of ICIs is similar in patients with *KRAS*-mutated and *KRAS*-nonmutated NSCLC (7). Hence, the relationship between oncogenic *KRAS* and tumor immunogenicity is of interest, particularly as selective KRAS inhibitors have now entered the clinical arena (9). These agents have demonstrated an immune stimulating effect in preclinical study (10) and are now approved as monotherapy in the second-line treatment setting and under clinical investigation in combination with ICIs (11).

However, despite seemingly conferring sensitivity to ICIs, oncogenic *KRAS* mutations are associated with poor prognosis in patients with NSCLC (12). There is emerging evidence suggesting that the prognostic value of *KRAS* mutations are modified by the presence of several co-occurring mutations, most notably *TP53* and *STK11*, the two most commonly co-mutated genes in KRAS-driven NSCLC (13, 14). Patients with concomitant *KRAS*/*TP53* mutations have comparatively shorter overall survival (OS) than patients with only a *KRAS* or *TP53* mutation (15). Yet, recent evidence also suggests that *KRAS*/*TP53* co-mutated patients are highly sensitive to ICIs, showing significant improvements in both objective response rates and progression-free survival compared with those with either *KRAS* or *TP53* mutations (8). Combined *KRAS*/*STK11* mutations are also associated with a poor prognosis (16, 17), as well as diminished sensitivity to ICIs (18, 19). Although the prognostic role of *STK11* mutations is well-established and their role on ICI responsiveness is emerging, the mechanisms through which these mutations alter tumor immunogenicity is unknown.

In the present study, we evaluated the immunogenomic landscape of a highly diverse group of 189 patients with NSCLC. In our cohort, oncogenic *KRAS* mutations were associated with increased CD8^+^ T-cell infiltration; however, this association was lost in patients with *STK11* co-mutation. Interestingly, *KRAS-*associated immunogenicity was strongly associated with an increase in lipid metabolism, which was lost in the *KRAS/STK11* co-mutated group. Similarly, *KRAS*-mutated tumors with robust expression of lipid metabolism genes were highly immunogenic and associated with improved OS, whereas those with low expression of lipid metabolism genes had an immune-excluded phenotype and were associated with shorter OS. Hence, these data offer insights into the potential mechanism through which *STK11* mutation suppresses *KRAS*-induced immunogenicity, namely, through the inhibition of lipid metabolism. Hence, alterations to lipid metabolism warrant consideration as a predictive biomarker and potential target for therapy in patients with *KRAS*-mutated NSCLC.

## Materials and Methods

### Patients, follow‐up, and sample collection

All tissues were collected from patients more than 18 years of age with NSCLC and sent to Tempus Labs for standard-of-care comprehensive molecular testing from March 2021 to April 2023. Patients were consented for comprehensive molecular testing, as is standard practice. Patients were initially evaluated during an outpatient consult, including a clinical examination and collection of basic demographic information. Paraffin-embedded specimens from each patient were sent for standardized histopathology and genomic analysis as described below. All patients were treated with standard of care per the National Comprehensive Cancer Network guidelines, and clinicopathologic and survival data were collected for all patients.

### Exclusion criteria

Patients were excluded if they had received a prior line of therapy or lost to follow-up.

### Histology and IHC

Tissues were fixed in 10% formalin and paraffin embedded. Following definitive diagnosis from the Department of Pathology, blocks were sent to Tempus Labs (Tempus Labs Inc.) where they were sectioned at 4-μm interval and stained with either hematoxylin and eosin or via IHC for PDL1 using the 22C3 anti-PDL1 antibody (Dako). PDL1 stains were quantified using the PDL1 tumor proportion score (TPS) as described by the manufacturer. For more details about PDL1 staining and interpretation, please refer to the current version of the PDL1 IHC 22C3 pharmDx Instructions for Use for PDL1 IHC 22C3 pharmDx, Code SK006.

### DNA/RNA sequencing

DNA sequencing was performed using the previously described Tempus xT (GTR Test IDHelp: GTR000558436.11) or xF (GTR Test IDHelp: GTR000569040.5) assays (Tempus Labs Inc.). RNA sequencing (RNA-seq) was performed using the Tempus xR assay. The Tempus xR panel quantifies transcript and gene level expression and identifies transcriptional evidence of chromosomal rearrangements that result in the expression of fusion RNA species. xR spans a 39-Mb target region of the human genome and covers 51 Mb of end-to-end tiled probe space.

Total nucleic acid is extracted from tissue specimens using Chemagic 360 or similar instrumentation. Recovered total nucleic acid is then quantified and qualified. RNA is fragmented using heat and magnesium to yield similar sized fragments from RNA inputs with different starting size distributions. Amplified target-captured libraries are pooled and sequenced on an Illumina HiSeq 4000 system using patterned flow cell technology to a targeted minimum depth of 30 million reads per sample. Strand-specific library preparation is performed using the KAPA RNA HyperPrep Kit for Illumina. Libraries are amplified with high fidelity, low-bias PCR using primers complementary to adapter sequences. Amplified libraries are subjected to a 1× magnetic bead–based cleanup to eliminate unused primers, and the quantity is assessed.

Following library preparation and amplification, targets are captured by hybridization, clean-up of captured targets is performed, and unbound fragments are washed away. Library capture is conducted using the xGen Exome Research Panel v1 probe set, along with the xGen Hybridization and Wash Kit and xGen Universal Blockers. RNA-seq data are then analyzed using the Tempus bioinformatics pipeline, with expression profiling using Kallisto and Fusion calling via their in-house method using GRCh37 reference. The bioinformatics pipeline includes a combination of software developed by Tempus as well as open-source and proprietary software applications developed by third parties.

The proportion of immune cells compared with tumor and stroma cells, as well as the relative proportions of key immune subtypes, is estimated using gene expression data from RNA-seq. The Tempus immune infiltration algorithm estimates the relative proportion of immune subtypes using a support vector regression (SVR) model, which includes an L2 regularizer and an epsilon-insensitive loss function, similar to the Newman and colleagues protocol (20). The SVR was implemented in Python using the nuSVR function in the SVM library of scikit-learn (0.18), with the LM22 reference matrix downloaded from the initial Newman and colleagues report (20). Cell populations were also estimated using the xCell version 1.1.0 package as described in the original reference (21). Additionally, CD cytotoxicity scores were calculated for each patient and derived from the expression levels of key cytotoxicity-associated genes, including *GZMA*, *GZMB*, *GZMH*, *GZMK*, *GZMM*, and *PRF1* (22, 23).

### Gene set enrichment analysis

Patients were arranged into groups as described, and values from RNA-seq data determined using the average log_2_(TPM + 1) values for each group. Using these values, sequencing data was subjected to gene set enrichment analysis. In brief, we downloaded the gmt file for C5:GO (Gene Ontology) biological process from MSigDB collections (Human MSigDB v2023.2.Hs updated October 2023; refs. 24, 25), restricting our analysis to include only datasets with a minimum of 20 members. Using Gitools (26) software, we selected for *Z*-score with a sampling size of 10,000. Group means was used as the estimator and Benjamini–Hochberg FDR as the multiple test correction. Heatmaps were generated using the *pheatmap* package (Version 1.0.12) in R (27). Correlation matrices between the expression of different genes were obtained using the *cor* function in R (27).

### Statistical analysis

Data were analyzed by either student *t* test or ANOVA fit to a general linear model in Minitab express, the validity of which was tested by adherence to the normality assumption, and the fitted plot of the investigator-assessed OS was measured from the time of diagnosis to either death or censoring, which was defined as the date of analysis. OS was reported using the Kaplan–Meier method/log-rank test with hazard ratios and considered significant at a *P* value of < 0.05 as described previously (28–32).

### De-identification and data storage

De-identified raw FASTQ sequencing data were obtained from Tempus as described below and stored in a secure server with password encryption. Retrospective data analysis was performed on deidentified data after approval by an Institutional Review Board (IRB 2022-0705) and in accordance with the U.S. Department of Health and Human Services and the Declaration of Helsinki. A waiver was granted for informed consent because of the retrospective nature of the study. De-identification of data was maintained through all steps and generic identifiers were used to notate and analyze data.

### Study approval

As described, de-identified RNA-seq data was obtained as part of standard of care comprehensive molecular testing through Tempus laboratories, and matched to clinical, demographic, and outcomes data following local IRB approval from the University of Illinois at Chicago (IRB 2022-0705). All patients provided written informed consent to participate, and all studies were conducted in accordance with recognized ethical guidelines (e.g., Declaration of Helsinki, CIOMS, Belmont Report, and US Common Rule). As above, this study was approved by the IRB at the University of Illinois at Chicago under protocol number IRB 2022-0705.

### Data availability

For the UI Health cohort, the data that support the findings of this study are available on request. The data are not publicly available as the subjects were not consented for the publication or distribution of their raw sequencing data. For additional information, please contact the corresponding author.

## Results

### Genetic landscape of cohort of patients with NSCLC

**Figure 1:**
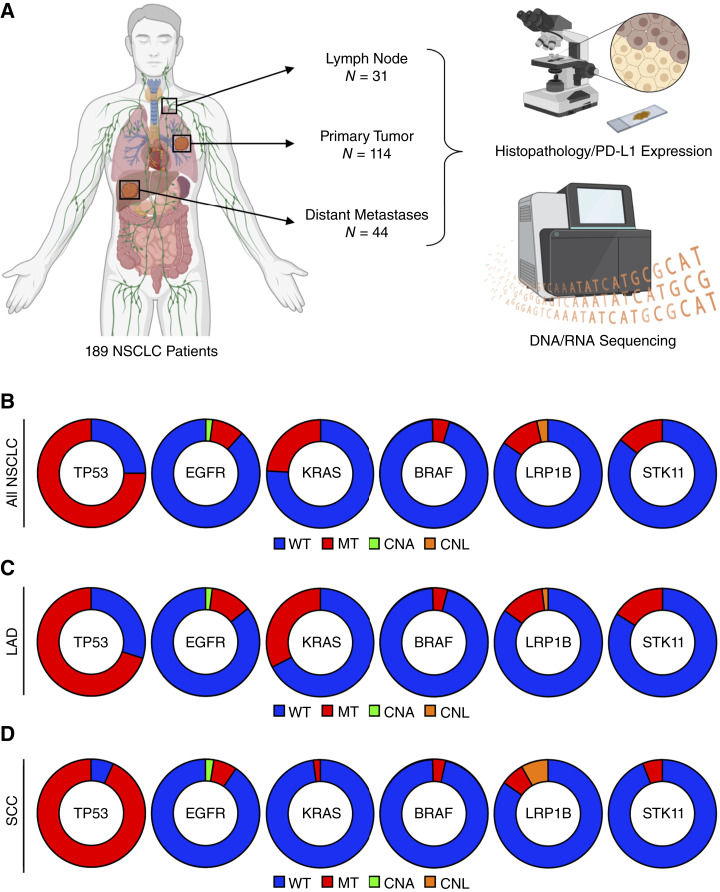
Genetic landscape of cohort of patients with NSCLC. **A,** Tissue biopsies from 189 treatment-naïve patients with NSCLC, 114 of which were from the primary lung tumor, 31 from an affected lymph node, and 44 from a metastatic site, were sectioned and stained with either hematoxylin and eosin or via IHC for PDL1. Tissues then underwent a standardized genomic analysis consisting of whole-exome DNA sequencing and whole-transcriptome RNA-seq. Pie charts are shown representing the most frequently observed mutations across (**B**) all 189 patients, (**C**) the 132 patients with LUAD, and (**D**) the 57 patients with SCC. CNA, copy-number amplification; CNL, copy-number loss; MT, mutant.

To explore the association between the genetic landscape and tumor immune microenvironment, we enrolled a total of 189 patients with NSCLC (Table 1). Of these patients, 132 were diagnosed with lung adenocarcinoma (LUAD) and the remaining 57 with squamous cell carcinoma (SCC). Diagnostic biopsies were obtained for each patient, 114 of which were from the primary lung tumor, 31 from an affected lymph node, and the remaining 44 from a metastatic site. These paraffin-embedded specimens underwent standardized genomic analyses including whole-exome DNA sequencing and whole-transcriptome RNA-seq (Fig. 1A). Across the entire cohort, *TP53* was the most commonly altered gene with 145 (76.7%) patients harboring a loss-of-function mutation. Gain-of-function *KRAS* mutations were the second most common alteration, affecting 44 (23.3%) patients. Deleterious mutation and/or copy-number loss of *STK11* or *LRP1B* were also common, affecting 29 (15.3%) and 27 (14.3%) patients, respectively. Gain-of-function mutations or copy-number amplification of *EGFR* were observed in 29 (12.2%) patients, and those affecting *BRAF* in eight (4.2%) patients (Fig. 1B). The distributions of these mutations were similar across both cancer histologies, with the exception of oncogenic *KRAS* mutations, which were almost exclusive to LUAD apart from a single patient with squamous cell carcinoma with a *KRAS*^*G12C*^ mutation (Fig. 1C and D).

**Table 1:** Clinical and demographic characteristics of the patient cohort

Age, years [*n* (%)]	
Median (range)	65 (31–93)
<50	12 (6.3%)
50–60	50 (26.5%)
61–70	74 (39.2%)
>70	53 (29%)
Natal sex, *n* (%)	
Male	97 (51.3%)
Female	92 (48.7%)
Race/ethnicity, *n* (%)	
Asian	15 (8.0%)
Black/African American	127 (67.1%)
Hispanic/Latino	17 (9.0%)
Non-Hispanic White/Caucasian	30 (15.9%)
Smoking status, *n* (%)	
Active	77 (40.7%)
Former	89 (40.1%)
Never	23 (12.2%)
Tumor stage, *n* (%)	
I	40 (21.2%)
II	17 (9.0%)
III	48 (25.4%)
IV	84 (44.4%)
Microsatellite status, *n* (%)	
MSS	187 (98.9%)
MSI-H	2 (1.1%)
ICI, *n* (%)	
None	99 (52.4%)
Pembrolizumab	63 (33.3%)
Atezolizumab	9 (4.8%)
Durvalumab	11 (5.8%)
Nivolumab	6 (3.2%)
Ipilimumab/nivolumab	1 (0.5%)

Abbreviations: MSI-H, microsatellite instability high; MSS, microsatellite stable.

### *KRAS*-mutated NSCLC tumors display increased PDL1 expression

**Figure 2:**
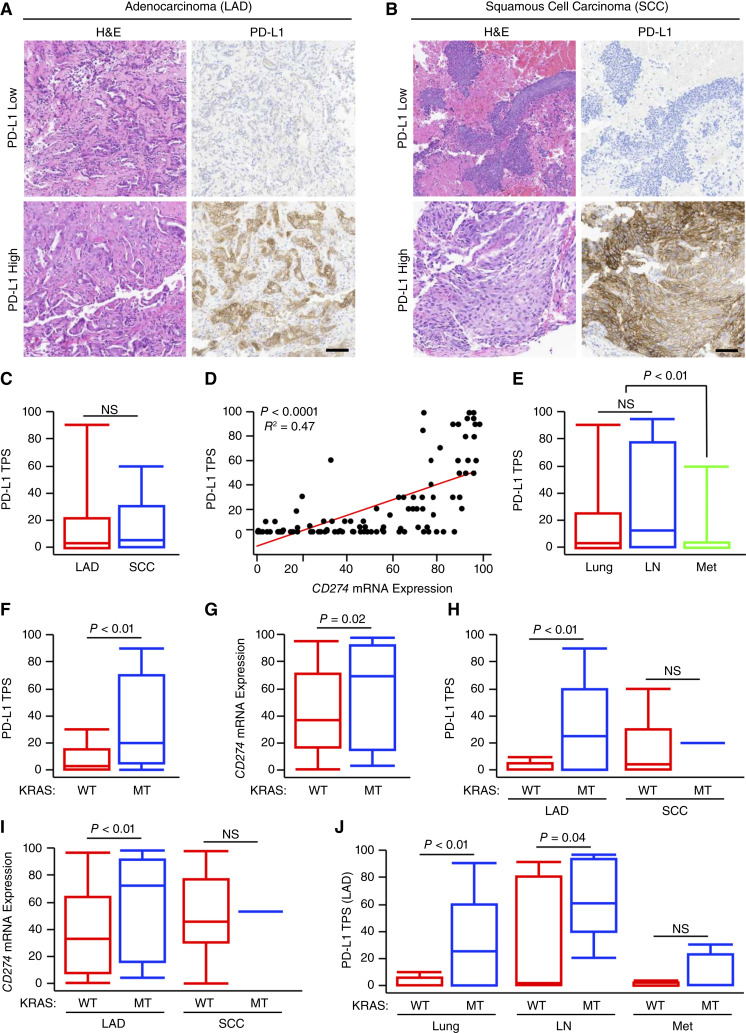
*KRAS*-mutated NSCLC tumors display increased PDL1 expression. **A** and **B,** Tissue biopsies from 189 treatment-naïve patients with NSCLC were stained with either H&E or via IHC for PDL1. Representative images are shown for low and high expression in LUAD (*N* = 132) and SCC (*N* = 57) histologies. **C,** PDL1 TPS arranged by tumor histology. **D,** PDL1 TPS plotted against percentile *CD274* mRNA expression. **E,** PDL1 TPS expression for tumor specimens obtained from the primary lung tumor (lung, *N* = 114), lymph node (LN, *N* = 31), or distant metastatic site (Met, *N* = 4). **F** and **G,** PDL1 TPS or *CD274* mRNA expression for either *KRAS*-WT (*N* = 144) or *KRAS* mutated patients (MT, *N* = 45). **H** and **I,** PDL1 TPS or *CD274* mRNA for either *KRAS*-WT or *KRAS*-MT patients arranged by tumor histology. **J,** PDL1 TPS or *CD274* mRNA for either *KRAS*-WT or *KRAS*-MT patients arranged by tumor site. H&E, hematoxylin and eosin; NS, nonsignificant.

In parallel to the above genomic analyses, tumor tissues were also stained via IHC for PDL1 expression (Fig. 2A and B). Although tumor histology had no significant effect on PDL1 TPS (Fig. 2C), there was a highly significant association between PDL1 TPS and mRNA expression of the PDL1 transcript *CD274* (Fig. 2D). PDL1 TPS was similar between primary tumor and lymph node specimens; however, distant metastases had significantly lower PDL1 expression (Fig. 2E), as reported previously (33). Interestingly, patients with an oncogenic *KRAS* mutation had increased expression of PDL1 protein as well as *CD274* mRNA expression (Fig. 2F and G). As *KRAS* mutations were largely unique to patients with LUAD, we next explored the relationship between *KRAS* mutation status and PDL1 expression while controlling for tumor histology. As previously, *KRAS*-mutated LUAD tumors had a highly significant increase in both PDL1 TPS as well as *CD274* mRNA expression, though this was not observed in squamous cell carcinoma presumably because of the relative lack of *KRAS* mutations (Fig. 2H and I). The relationship between *KRAS* mutation and PDL1 expression was independent of tumor site, though the effect in metastatic tumors did not reach statistical significance because of the small size of this group and low frequency of *KRAS* mutations (Fig. 2J).

### *STK11* co-mutation negates *KRAS*-induced immunogenicity

**Figure 3:**
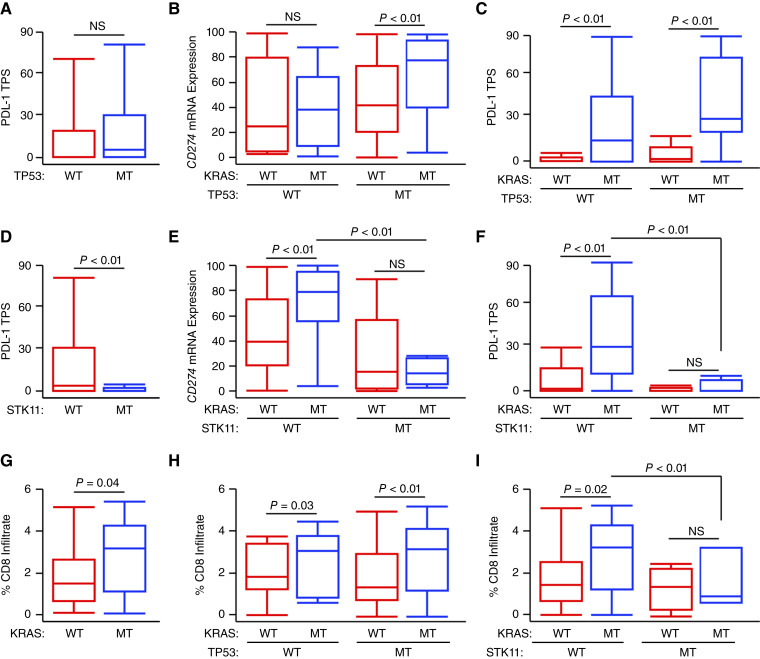
*STK11* co-mutation negates *KRAS*-induced immunogenicity. **A,** PDL1 TPS for all patients with NSCLC arranged by *TP53* mutation status [*N* = 44 WT; *N* = 145 *TP53*-mutated (MT)]. **B** and **C,***CD274* mRNA expression or PDL1 TPS arranged by combined *KRAS* and *TP53* mutation statuses (*N* = 26 *KRAS*-WT/*TP53*-WT, *N* = 18 *KRAS*-MT/*TP53*-WT, *N* = 118 *KRAS*-WT/*TP53*-MT, *N* = 27 *KRAS*-MT/*TP53*-MT). **D,** PDL1 TPS arranged by *STK11* mutation status (*N* = 166 wild-type or WT, *N* = 23 *STK11*-mutated or MT). **E** and **F,***CD274* mRNA expression or PDL1 TPS arranged by combined *KRAS* and *STK11* mutation statuses (*N* = 131 *KRAS*-WT/*STK11*-WT; *N* = 35 *KRAS*-MT/*STK11*-WT; *N* = 13 *KRAS*-WT/*STK11*-MT; *N* = 10 *KRAS*-MT/*STK11*-MT). **G,** Percent CD8^+^ T-cell infiltration arranged by *KRAS* mutation status. **H,** Percent CD8^+^ T-cell infiltration arranged by combined *KRAS* and *TP53* mutation statuses. **I,** Percent CD8^+^ T-cell infiltration arranged by combined *KRAS* and *STK11* mutation statuses.

Though *KRAS*-mutated NSCLC tumors displayed increased mean PDL1 expression in our cohort, it is important to note that PDL1 expression is highly variable across *KRAS*-mutated patients. We therefore explored the effect of co-mutations in other commonly altered genes in our cohort on PDL1 expression as well as corresponding changes in the tumor immune infiltrate. As reported (34), *KRAS* and *EGFR* mutations were mutually exclusive, as were *KRAS* and *BRAF* mutations. Hence, neither *EGFR* nor *BRAF* mutation influenced the association between *KRAS* mutation status and either *CD274* mRNA expression or PDL1 TPS, nor did *LRP1B* mutation status (Supplementary Fig. S1A–S1F). However, although *TP53* mutation alone was not associated with a significant change in PDL1 TPS (Fig. 3A), *TP53* mutation status significantly modified the association between *KRAS* mutation status and *CD274* mRNA expression. In *TP53*-nonmutated patients, there was no association between *KRAS* mutation status and *CD274* mRNA expression (Fig. 3B). However, this was not reflected by corresponding changes in PDL1 protein expression, in which *KRAS*-mutated tumors were associated with PDL1 TPS independent of *TP53* mutation status (Fig. 3C). Interestingly, patients with loss-of-function *STK11* mutations had a significant decrease in PDL1 TPS across the entire cohort (Fig. 3D). In *STK11* wild-type (WT) patients, *KRAS*-mutated tumors had highly significant increases in both mRNA expression of *CD274* as well as in PDL1 TPS. However, in *STK11*-mutated tumors, *KRAS* mutation status had no relationship to either *CD274* mRNA expression or PDL1 TPS (Fig. 3E and F).

In addition to increased PDL1 TPS, *KRAS*-mutated tumors also had a significant increase in CD8^+^ T-cell infiltration determined by the Tempus immune infiltration algorithm (Fig. 3G), particularly those with a KRAS^G12^ mutation (Supplementary Fig. S2). There was no relationship between *KRAS* mutation status and either tumor mutational burden or the infiltration of other immune cell subpopulations via the Tempus algorithm (Supplementary Fig. S3). In patients harboring a deleterious *TP53* mutation, the relationship between *KRAS* mutation status and CD8^+^ T-cell infiltration was highly significant, though this was more modest in the *TP53*-WT group (Fig. 3H). However, in patients with either a loss-of-function *STK11* or *LRP1B* mutation, *KRAS* mutations had no effect on CD8^+^ T-cell infiltration (Fig. 3I; Supplementary Fig. S4).

As clinical data suggest that patients with combined *KRAS* and *STK11* mutations may be poorly sensitive to ICIs (18, 19), we next estimated immune and stroma cell populations using the xCell deconvolution algorithm. In addition to re-demonstrating a CD8^+^ T-cell excluded phenotype in the *KRAS*/*STK11* co-mutated group, this also suggested that *KRAS*/*STK11* co-mutated tumors have a relative decrease in CD4^+^ T-cells, B-cell infiltration, dendritic cells, and fibroblasts, and display a relative increase in eosinophils (Supplementary Fig. S5). To further evaluate potential changes in cell-mediated cytotoxicity, we computed the CD cytotoxicity score based on *KRAS* and *STK11* mutation status, again showing an increase in *KRAS*-mutant, *STK11*-WT patients, and a decrease in *KRAS* and *STK11* co-mutated tumors (Supplementary Fig. S6).

### *KRAS*-induced immunogenicity is associated with increased lipid metabolism

**Figure 4:**
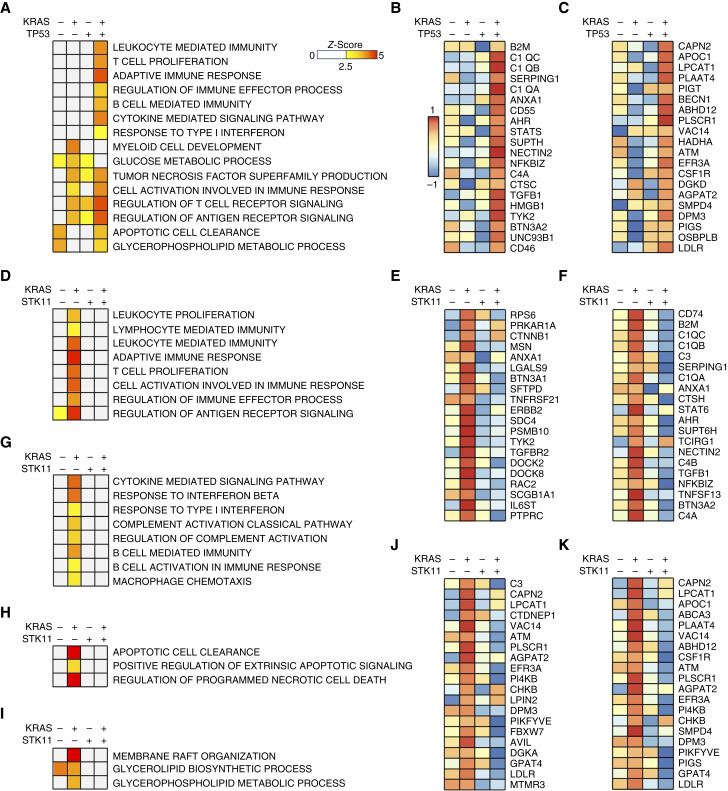
Mutant *KRAS*-induced immunogenicity is associated with increased lipid metabolism. **A,** Tissue biopsies from treatment-naïve patients with NSCLC were subjected to whole-transcriptome RNA-seq. Patients were grouped based on *KRAS* and *TP53* mutation statuses and gene set enrichment analysis (GSEA) performed. Significantly altered gene sets involved in tumor immunity, apoptosis, or metabolism are shown using a *Z*-score cutoff of 2.5. Focused heatmaps are shown for select, significantly altered genes in the (**B**) adaptive immune response gene set or (**C**) glycerophospholipid metabolic process gene sets, both using a FDR-adjusted *P* value of <0.05. **D,** Patients were grouped based on *KRAS* and *STK11* mutation statuses, and GSEA performed as previously and significantly altered gene sets involved in the adaptive immune response shown. Focused heatmaps are shown for select, significantly altered genes in the (**E**) lymphocyte-mediated immunity gene set or (**F**) cell activation involved in immune response gene sets, both using a FDR-adjusted *P* value of <0.05. Patients were again grouped based on *KRAS* and *STK11* mutation statuses and GSEA performed. Significantly altered gene sets are shown for pathways involved in **G** innate or humoral immunity, (**H**) apoptosis/cell death, or (**I**) lipid metabolic processes. Focused heatmaps are shown for select, significantly altered genes in the (**J**) glycerolipid biosynthetic process gene set or (**K**) glycerophospholipid metabolic process gene set, both using a FDR-adjusted *P* value of <0.05.

Although our above observations suggest that loss of *TP53* may enhance mutant *KRAS*-induced immunogenicity and the loss of *STK11* may attenuate mutant *KRAS*-induced immunogenicity, the mechanism underlying these events are unclear. Given the small number of patients with concomitant *KRAS* and *LRP1B* mutation, we next grouped patients by *KRAS* and either *TP53* or *STK11* mutation status and compared changes in gene expression by gene set enrichment analysis using Gene Ontology biological process gene sets. In the *KRAS/TP53* co-mutated group, we observed significant upregulation of several immune cell processes compared with all other groups, most notably those involved in T-cell proliferation, the adaptive immune response, antigen receptor signaling, and cytokine production (Fig. 4A and B). Interestingly, this was associated with a relative decrease in glucose metabolic processes and a decrease in glycerophospholipid metabolic processes compared with the single-mutant groups (Fig. 4A–C).

Similarly, the *KRAS*-mutated, *STK11*-nonmutated group had significant upregulation of several gene sets involved in the adaptive immune response, lymphocyte activation/proliferation, and antigen-receptor signaling, as well as those involved in humoral immunity, macrophage chemotaxis, and cytokine production (Fig. 4D–G). This was associated with highly significant increases in several gene sets involved in apoptosis, suggestive of a functional antitumor immune response (Fig. 4H). However, in the *KRAS/STK11* co-mutated group, none of these gene sets were increased from the control group and all were downregulated compared with the *KRAS*-mutated, *STK11*-nonmutated group (Fig. 4D–H). Interestingly, in the immunologically active *KRAS*-mutated, *STK11*-nonmutated group, there was again a significant increase in several genes involved in lipid metabolism, namely, those in the membrane raft organization, glycerolipid synthesis, and glycerophospholipid metabolic processes (Fig. 4I–K). However, there was no increase in expression in any of these gene sets in the immune-excluded *KRAS/STK11* co-mutated group (Fig. 4I), with most lipid metabolism genes displaying significant downregulation compared with all other groups (Fig. 4J and K).

### Mutant *KRAS*-induced immunogenicity requires the transcriptional upregulation of lipid metabolism

**Figure 5:**
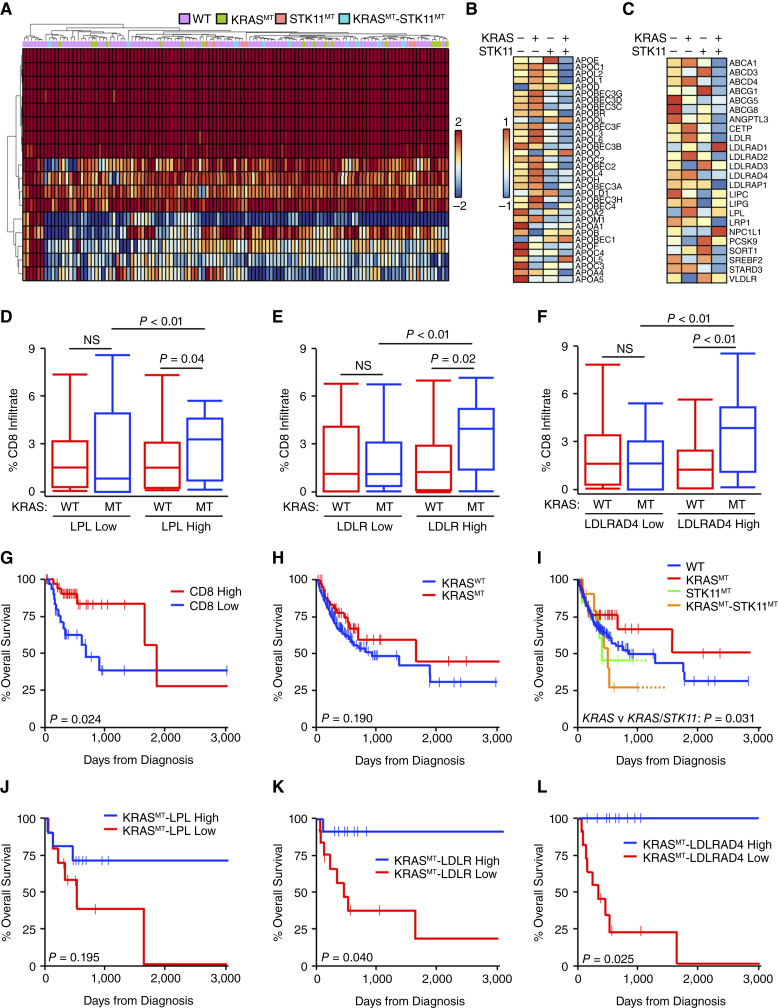
Mutant *KRAS*-induced immunogenicity requires the transcriptional upregulation of lipid metabolism. **A,** Tissue biopsies from treatment-naïve patients with NSCLC were subjected to whole-transcriptome RNA-seq as described and representative heat maps shown for select genes involved in lipid metabolism. **B** and **C,** Focused heatmaps are shown for select, significantly altered genes involved in lipid metabolic processes using a FDR-adjusted *P* value of <0.05. The percent CD8^+^ T-cell infiltration for patients arranged by combined *KRAS* mutation and (**D**) *LPL* mRNA expression, (**E**) *LDLR* mRNA expression, or (**F**) *LDLRAD4* mRNA expression. Kaplan–Meier plots indicating months of OS for patients with NSCLC arranged by (**G**) *CD8* mRNA expression, (**H**) *KRAS* mutation status alone, (**I**) combined *KRAS* and *STK11* mutation statuses, (**J**) KRAS mutation status and *LPL* mRNA expression, (**K**) KRAS mutation status and *LDLR* mRNA expression, and (**L**) KRAS mutation status and *LDLRAD4* mRNA expression. High, above median; low, below median; MT, mutated; NS, nonsignificant.

As mutated *KRAS*-associated immunogenicity seemed to correlate with corresponding increases in lipid metabolism, we next explored the expression of a wider range of lipid metabolism genes in relation to both *KRAS* and *STK11* mutation status. Several lipid metabolism genes not represented in the prior gene sets were also significantly downregulated in the *KRAS/STK11* co-mutated group, most notably those encoding for apolipoproteins and low-density lipid receptors (LDLR; Fig. 5A–C). Given the strong correlation between several of these genes and *CD8A* mRNA expression (Supplementary Fig. S7), we next explored whether the expression of several lipid metabolism genes also modified mutant *KRAS*-induced CD8^+^ T-cell infiltration. In patients with low (below median) mRNA expression of *lipoprotein lipase* (*LPL*), *KRAS* mutations had no effect on CD8^+^ T-cell infiltration. However, in patients with high (above median) *LPL* mRNA expression, *KRAS* mutations were associated with increased CD8^+^ T-cell infiltration (Fig. 5D). We observed similar but more statistically significant results related to *LDLR* mRNA expression (Fig. 5E) as well as *LDLRAD4* mRNA expression (Fig. 5F).

Given the above changes in CD8^+^ T-cell infiltration, we next sought to determine whether expression of these lipid metabolism genes had an effect on OS in our cohort. Expectedly, patients diagnosed with early-stage disease had a significant survival advantage compared with those diagnosed at more advanced stages (Supplementary Fig. S8A and S8B). Although patients with PDL1-positive tumors (TPS > 1) had a modest survival advantage (Supplementary Fig. S8C), as previously reported (35), patients with high (above median) *CD8A* mRNA expression had a highly significant OS benefit compared with those with low (below median) expression (Fig. 5G). Although *KRAS* mutation alone did not have a relationship to OS (Fig. 5H), patients with *KRAS*-mutated, *STK11*-WT tumors had a significant survival advantage compared with those with *KRAS/STK11* co-mutation (Fig. 5I). In patients harboring a *KRAS* mutation, those with high mRNA expression of *LPL* had improved OS compared with those with low *LPL* expression (Fig. 5J). Similarly, those with high expression of *LDLR* and *LDLRAD4* also had a significant survival advantage compared with those with low expression (Fig. 5K and L).

## Discussion

Immunotherapy has transformed the treatment paradigm for several solid tumors (36–42). Accordingly, ICI-based immunotherapy is now the cornerstone of treatment for many patients with NSCLC (43). Although many patients treated with ICIs achieve durable immune responses and derive long-term clinical benefit, others are poorly ICI-sensitive and fail to derive any meaningful survival benefit (44). Hence, there is an increasing interest in identifying both predictive biomarkers for ICI sensitivity, as well as mechanism-driven combination strategies to improve the therapeutic efficacy of ICIs. Recent evidence suggests that the genomic landscape of NSCLC tumors is a key factor in both overall prognosis and ICI responsiveness (45). For instance, though gain-of-function *KRAS* mutations are associated with poor prognosis in NSCLC (12), there are emerging data suggesting that *KRAS*-mutated NSCLC tumors are preferentially sensitive to ICIs (46). However, this seems to be heavily influenced by the presence of select co-mutations, most notably loss-of-function mutations to *STK11* (46). Although the field has reached consensus about the poor prognosis associated with *KRAS*/*STK11* co-mutation (16, 17) and immune-excluded phenotype in these patients (18, 19), the mechanisms through which *STK11* loss attenuates mutant *KRAS*-induced immunogenicity are poorly understood.

To this end, metabolic reprogramming is a hallmark feature of tumorigenesis (47, 48). Classically, tumor metabolism has been defined by the shift from mitochondrial oxidative phosphorylation to aerobic glycolysis, a phenomenon known as the “Warburg effect” (49). However, it is now widely accepted that cancer-associated metabolic derangements are far more extensive, involving any number of additional cell processes (50). For instance, tumor cells undergo extensive rewiring of lipid uptake, storage, utilization, and synthesis (51). Recently, these changes in metabolism have been shown to play important roles in regulating the tumor immune microenvironment and dictating responses to cancer immunotherapy (52, 53). To this end, *LKB1*, the gene product of *STK11*, is emerging as a central regulator of tumor metabolism. The seminal work exploring the role of LKB1 in metabolism was performed in murine models of Peutz–Jeghers syndrome, a familial cancer syndrome driven by germline loss-of-function *STK11* mutations. In this study, *Stk11* haploinsufficient mice developed extensive gastrointestinal hamartomas, similar to those observed in patients with Peutz–Jeghers syndrome. These hamartomas were increasingly 18F-deoxyglucose-PET avid, suggesting that *Stk11*/LKB1 loss increases tumor cell uptake of glucose (54). The authors further demonstrated that this phenomenon was driven by mTORC1-mediated overexpression of HIF1α and ameliorated by rapamycin (54).

Similarly, lung cancer cells with loss of LKB1 displayed increased uptake and utilization of both glucose and glutamine, supporting ATP production and macromolecular biosynthesis (55). This was again dependent on HIF1α signaling and was antagonized by rapamycin (55). In mutant *KRAS*-driven NSCLC cells, LKB1 loss resulted in increased redox stress, increased levels of intracellular reactive oxygen species, and decreased ATP synthesis, NADPH/NADP+ ratio, and glutathione levels (56). This was associated with increased glutamine dependence and vulnerability to pharmacologic inhibition of glutaminase (56). Pharmacologic inhibition of glutaminase has since been shown to sensitize LKB1-deficient NSCLC cells to ionizing radiation (57). These and other studies suggest that LKB1 functions as a negative regulator of the Warburg effect and that *STK11*-mutated tumors are more dependent on anaerobic glycolysis (58). This is paralleled by a reduction in fatty acid synthesis in cells lacking LKB1, thereby protecting tumor cells from lipid hydroperoxide accumulation and ferroptosis (59).

*KRAS* itself has also been linked to energy dysregulation, including alterations to lipid metabolism. In pancreatic cancer cells, *KRAS*^G12D^-dependent upregulation of hormone-sensitive lipase has been shown to bias cells toward increased lipid storage (60). Similarly, in lung tumor cells, *KRAS* drives ERK-mediated expression of fatty acid synthase to increase *de novo* lipogenesis (61). Also in lung cancer, oncogenic *KRAS* allows for irreversible MYC overexpression, thereby driving cytosolic phospholipase A2 activity to release membrane-bound arachidonic acid and activate lipoxygenase and cyclooxygenase pathways (62). Given the proinflammatory roles of lipoxygenase and cyclooxygenase signaling, this is a plausible mechanism through which *KRAS*-induced lipid metabolism may augment tumor immunogenicity in our cohort, which may be ameliorated upon *STK11* mutation and the presumptive shift of tumor cell metabolism away from lipolysis and toward anaerobic glycolysis.

Although this has yet to be conclusively linked to lung cancer immunogenicity, there is a growing body of data suggesting that modifying lipid metabolism can have dramatic effects on the tumor immune program. For instance, fibrates have been shown to augment responses to ICIs in both preclinical studies as well as in patients with NSCLC (63–65). Several studies have also demonstrated that statins modify the immune microenvironment and potentiate ICIs in several cancer histologies including NSCLC (66–69). Importantly, these studies do not account for *KRAS* or *STK11* mutation status. Hence, future studies would benefit from careful evaluation of genetic subgroups to identify those who would most benefit from these or similar approaches. Additionally, our study was conducted in a highly diverse cohort, with most patients belonging to minority groups. As the genetic landscape of patients with lung cancer can vary significantly based on ethnicity (70) and minority groups are often unrepresented in large-scale sequencing studies (71), our findings underscore the importance of minority inclusion in immunogenomic studies.

**Figure 6:**
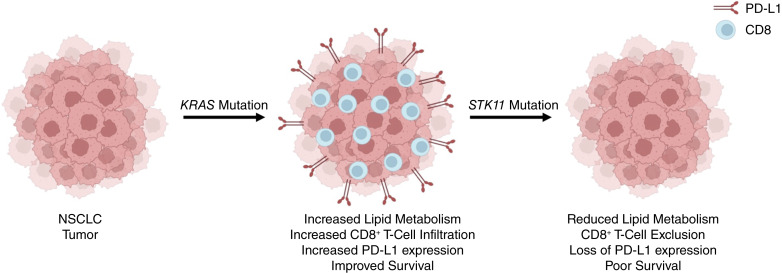
Schema describing the presumptive mechanism through which STK11 loss attenuates KRAS-induced immunogenicity in NSCLC. In patients with NSCLC, gain-of-function *KRAS* mutations seem to enhance tumor immunogenicity, increasing both PDL1 expression and CD8^+^ T-cell infiltration. However, *KRAS*/*STK11* co-mutated tumors displayed an immune-excluded phenotype, with diminished expression of PDL1 and limited CD8^+^ T-cell infiltration. This immune-excluded phenotype was paralleled by reductions in lipid metabolism, suggesting that the loss of *STK11* may function as a metabolic switch, suppressing lipid metabolism in favor of glycolysis to attenuate *KRAS*-induced immunogenicity.

Although our findings are interesting, our study has inherent limitations that must be considered. For instance, though these 189 patients took many years to accrue, the relatively small sample size precludes us from performing more in-depth analyses that may allow for additional mechanistic insights. Hence, as with all single center studies, our findings should be validated in a larger cohort. Additionally, as is the nature of retrospective data, our findings are largely correlative. Hence, our findings also warrant careful laboratory exploration to more conclusively link *KRAS/STK11* mutations, lipid metabolism, and tumor immunity. Despite these limitations, our data suggests that mutant *KRAS*-induced immunogenicity in NSCLC may require an *STK11*/LKB1-dependent shift toward lipid metabolism (Fig. 6). Although further mechanistic studies and validation in a larger cohort are required, this may provide both a potential biomarker for ICI sensitivity as well as a potential target for therapy, particularly given the immune stimulating effects of commonly used lipid modifying medications.

## Supplementary Material

Supplemental Figure Legends Supplemental Figure Legends

Figure S1 Figure S1. KRAS-induced PD-L1 expression is unmodified by EGFR, BRAF, or LRP1B status

Figure S2 Figure S2. KRASG12 mutations are associated with increased PD-L1 expression and CD8+ T-cell infiltration

Figure S3 Figure S3. KRAS mutation is not associated with additional alterations to tumor immunogenicity

Figure S4 Figure S4. KRAS-induced CD8+ T-cell infiltration is unmodified by EGFR or BRAF status, but lost in LRP1B co-mutated tumors

Figure S5 Figure S5. KRAS/STK11 co-mutated tumors have an immune excluded phenotype via the xCell deconvolution algorithm

Figure S6 Figure S6. KRAS mutated tumors have an increased CD cytotoxicity score, which is lost with STK11 co-mutation

Figure S7 Figure S7F. Correlation between genes involved in lipid metabolism and those involved in immune processes

Figure S8 Figure S8. Tumor stage and PD-L1 expression independently associate with overall survival
