# Supplementary material for: Loss of STK11 Suppresses Lipid Metabolism and Attenuates KRAS-Induced Immunogenicity in Patients with Non–Small Cell Lung Cancer
Source: Cancer Res Commun. 2024 Aug 30;4(8):2282–94. doi: 10.1158/2767-9764.CRC-24-0153 (PMC11362717; doi:10.1158/2767-9764.CRC-24-0153)
Supplement: Supplemental Figure Legends [file crc-24-0153_supplemental_figure_legends_suppsfl.docx]

**Supplement: Loss of *STK11* Suppresses Lipid Metabolism and Attenuates *KRAS*-Induced Immunogenicity in Patients with Non-Small Cell Lung Cancer**

Daniel R. Principe^1*^, Mary M. Pasquinelli^2^, Ryan H. Nguyen^3^, Hidayatullah G. Munshi^4,3,6^, Alicia Hulbert^7^, Alexandre F. Aissa^8^, and Frank Weinberg^5*^

^1^Department of Medicine, University of Wisconsin, Madison, WI; ^2^Division of Pulmonary, Critical Care, Sleep and Allergy, University of Illinois at Chicago, Chicago, IL; ^3^Division of Hematology and Oncology, University of Illinois Chicago and Translational Oncology Program, University of Illinois Cancer Center, Chicago IL; ^4^Department of Medicine, Feinberg School of Medicine, Northwestern University, Chicago, IL; ^5^The Robert H. Lurie Comprehensive Cancer Center, Chicago, IL; ^6^Jesse Brown VA Medical Center, Chicago, IL; ^7^Department of Surgery, University of Illinois Chicago, Chicago, IL; ^8^Division of Genetics, Department of Morphology and Genetics, Federal University of São Paulo, São Paulo, Brazil

Short Title: Loss of STK11 attenuates KRAS-induced immunogenicity

Pages 3

Figures 8

Words 376

*Correspondence to:

Frank Weinberg MD, PhD

Department of Medicine

University of Illinois College of Medicine

840 S Wood St, Ste 820

Chicago, IL 60612

Tel: (847)421-2241

E-mail: [fweinb1@uic.edu](mailto:fweinb1@uic.edu)

or

Daniel R. Principe MD, PhD

Department of Medicine

University of Wisconsin

1685 Highland Avenue

Madison, WI USA 53705

Tel: (847) 502-2355

Email: [drprincipe@wisc.edu](mailto:principe@uic.edu)

**Conflict of Interest Disclosure:** The authors have no conflicts to disclose.

**SUPPLEMENTAL FIGURE LEGENDS**

**Figure S1. *KRAS-induced PD-L1 expression is unmodified by EGFR, BRAF*, or *LRP1B* status**

CD274 mRNA expression arranged by **(A)** combined *KRAS* and *EGFR* mutation status, **(B)** combined *KRAS* and *BRAF* mutation status, or **(C)** combined *KRAS* and *LRP1B* mutation status. PD-L1 Tumor Proportion Score (TPS) arranged by **(D)** combined *KRAS* and *EGFR* mutation status, **(E)** combined *KRAS* and *BRAF* mutation status, or **(F)** combined *KRAS* and *LRP1B* mutation status. WT: wild type, MT: mutant.

**Figure S2. *KRAS^G12^ mutations are associated with increased PD-L1 expression and CD8+ T-cell infiltration***

**(A)** PD-L1 Tumor Proportion Score (TPS) or **(B)** percent CD8+ T-cell infiltration arranged by KRAS mutation type. WT: wild type.

**Figure S3. *KRAS mutation is not associated with additional alterations to tumor immunogenicity***

**(A,B)** Tumor mutational burden for all NSCLC patients shown as either mutations per megabase (m/MB) or percentile when compared to the Tempus genomic database arranged by *KRAS* mutation status. **(C)** The Tempus immune infiltration algorithm was used to estimate the total percent immune cell infiltration, **(D)** percent macrophage infiltration, **(E)** percent B-cell infiltration, **(F)** percent natural killer (NK) cell infiltration, **(G)** percent T-cell infiltration, and **(H)** percent CD4+ T-cell infiltration. NS: non-significant.

**Figure S4. *KRAS-induced CD8+ T-cell infiltration is unmodified by EGFR or BRAF* *status, but lost in LRP1B co-mutated tumors***

Percent CD8+ T-cell infiltration arranged by **(A)** combined *KRAS* and *EGFR* mutation status, **(B)** combined *KRAS* and *BRAF* mutation status, or **(C)** combined *KRAS* and *LRP1B* mutation status**.**

**Figure S5. *KRAS/STK11 co-mutated tumors have an immune excluded phenotype via the xCell deconvolution algorithm***

Immune and stromal cell types were estimated for patients using the xCell deconvolution algorithm and arranged by combined *KRAS* and *STK11* mutation status.

**Figure S6. *KRAS mutated tumors have an increased CD cytotoxicity score, which is lost with STK11 co-mutation***

CD cytotoxicity scores for patients arranged by combined *KRAS* and *STK11* mutation status.

**Figure S7. *Correlation between genes involved in lipid metabolism and those involved in immune processes***

Heatmap showing the Spearman’s correlation coefficient for select genes involved in either lipid metabolism or immune cell processes.

**Figure S8. *Tumor stage and PD-L1 expression independently associate with overall survival***

Kaplan–Meier plots indicating months of overall survival for NSCLC patients arranged by **(A,B)** tumor stage or **(C)** PD-L1 TPS.
