# Supplementary material for: Loss of STK11 Suppresses Lipid Metabolism and Attenuates KRAS-Induced Immunogenicity in Patients with Non–Small Cell Lung Cancer
Source: Cancer Res Commun. 2024 Aug 30;4(8):2282–94. doi: 10.1158/2767-9764.CRC-24-0153 (PMC11362717; doi:10.1158/2767-9764.CRC-24-0153)
Supplement: Figure S1 — KRAS-induced PD-L1 expression is unmodified by EGFR, BRAF, or LRP1B status [file crc-24-0153_figure_s1_supps1.pdf]

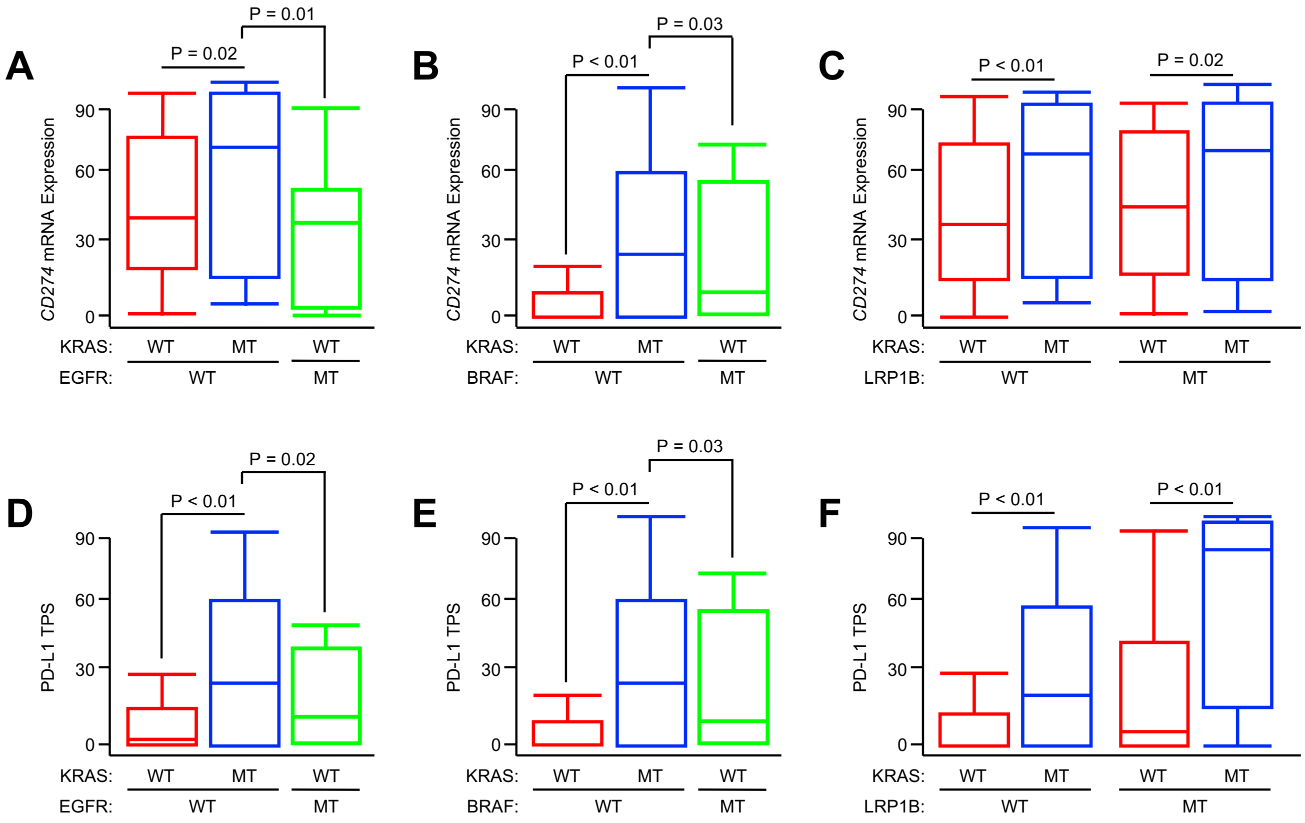

Figure S1. KRAS-induced PD-L1 expression is unmodified by *EGFR*, *BRAF*, or *LRP1B* status
CD274 mRNA expression arranged by **(A)** combined *KRAS* and *EGFR* mutation status, **(B)** combined *KRAS* and *BRAF* mutation status, or **(C)** combined *KRAS* and *LRP1B* mutation status. PD-L1 Tumor Proportion Score (TPS) arranged by **(D)** combined *KRAS* and *EGFR* mutation status, **(E)** combined *KRAS* and *BRAF* mutation status, or **(F)** combined *KRAS* and *LRP1B* mutation status. WT: wild type, MT: mutant.
