# Supplementary material for: Loss of STK11 Suppresses Lipid Metabolism and Attenuates KRAS-Induced Immunogenicity in Patients with Non–Small Cell Lung Cancer
Source: Cancer Res Commun. 2024 Aug 30;4(8):2282–94. doi: 10.1158/2767-9764.CRC-24-0153 (PMC11362717; doi:10.1158/2767-9764.CRC-24-0153)
Supplement: Figure S2 — KRASG12 mutations are associated with increased PD-L1 expression and CD8+ T-cell infiltration [file crc-24-0153_figure_s2_supps2.pdf]

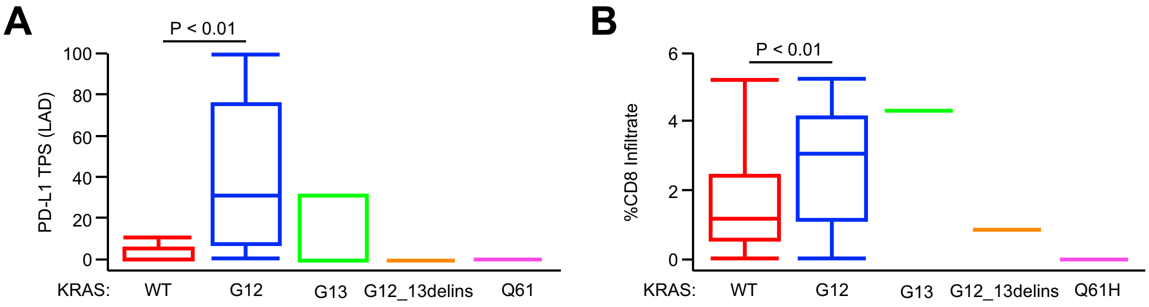

Figure S2. *KRAS*^{G12} mutations are associated with increased PD-L1 expression and CD8+ T-cell infiltration
(A) PD-L1 Tumor Proportion Score (TPS) or **(B)** percent CD8+ T-cell infiltration arranged by *KRAS* mutation type. WT: wild type.
