# Supplementary material for: Loss of STK11 Suppresses Lipid Metabolism and Attenuates KRAS-Induced Immunogenicity in Patients with Non–Small Cell Lung Cancer
Source: Cancer Res Commun. 2024 Aug 30;4(8):2282–94. doi: 10.1158/2767-9764.CRC-24-0153 (PMC11362717; doi:10.1158/2767-9764.CRC-24-0153)
Supplement: Figure S3 — KRAS mutation is not associated with additional alterations to tumor immunogenicity [file crc-24-0153_figure_s3_supps3.pdf]

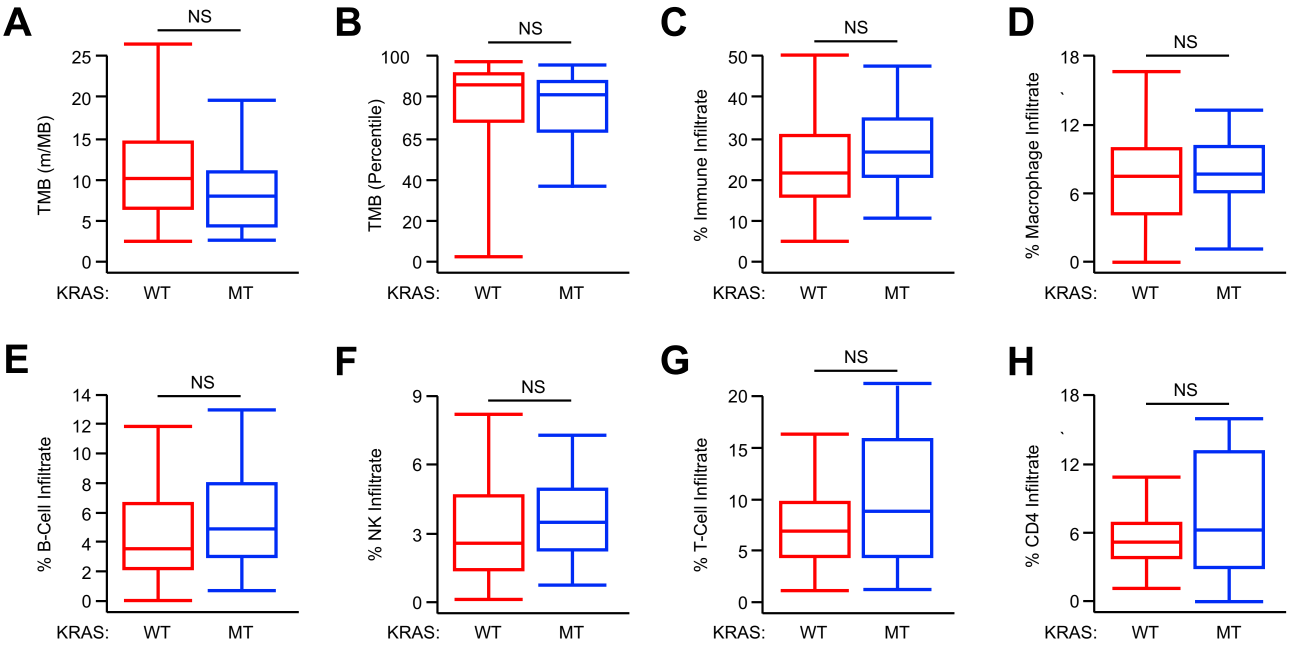

Figure S3. *KRAS* mutation is not associated with additional alterations to tumor immunogenicity
(A,B) Tumor mutational burden for all NSCLC patients shown as either mutations per megabase (m/MB) or percentile when compared to the Tempus genomic database arranged by *KRAS* mutation status. **(C)** The Tempus immune infiltration algorithm was used to estimate the total percent immune cell infiltration, **(D)** percent macrophage infiltration, **(E)** percent B-cell infiltration, **(F)** percent natural killer (NK) cell infiltration, **(G)** percent T-cell infiltration, and **(H)** percent CD4+ T-cell infiltration. NS: non-significant.
