# Supplementary material for: Loss of STK11 Suppresses Lipid Metabolism and Attenuates KRAS-Induced Immunogenicity in Patients with Non–Small Cell Lung Cancer
Source: Cancer Res Commun. 2024 Aug 30;4(8):2282–94. doi: 10.1158/2767-9764.CRC-24-0153 (PMC11362717; doi:10.1158/2767-9764.CRC-24-0153)
Supplement: Figure S4 — KRAS-induced CD8+ T-cell infiltration is unmodified by EGFR or BRAF status, but lost in LRP1B co-mutated tumors [file crc-24-0153_figure_s4_supps4.pdf]

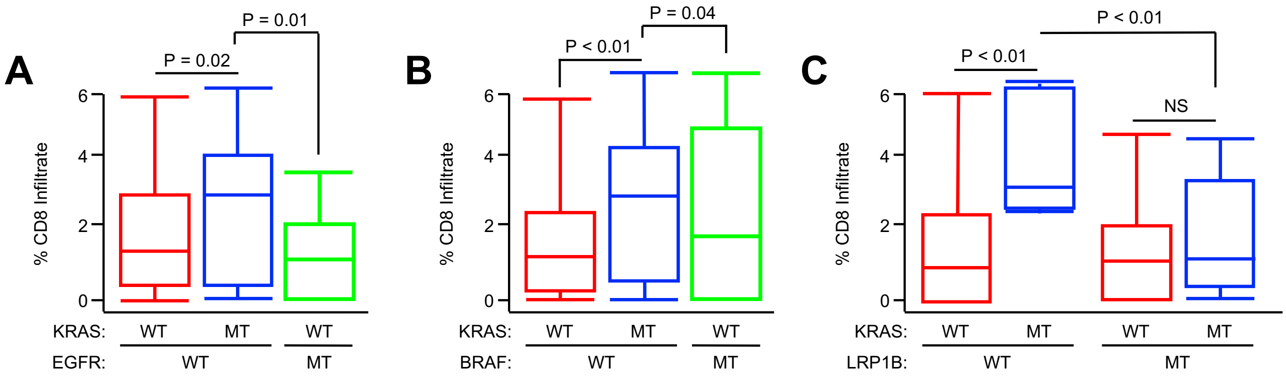

Figure S4. KRAS-induced CD8+ T-cell infiltration is unmodified by *EGFR* or *BRAF* status, but lost in *LRP1B* co-mutated tumors

Percent CD8+ T-cell infiltration arranged by **(A)** combined *KRAS* and *EGFR* mutation status, **(B)** combined *KRAS* and *BRAF* mutation status, or **(C)** combined *KRAS* and *LRP1B* mutation status**.**
