# Supplementary material for: Loss of STK11 Suppresses Lipid Metabolism and Attenuates KRAS-Induced Immunogenicity in Patients with Non–Small Cell Lung Cancer
Source: Cancer Res Commun. 2024 Aug 30;4(8):2282–94. doi: 10.1158/2767-9764.CRC-24-0153 (PMC11362717; doi:10.1158/2767-9764.CRC-24-0153)
Supplement: Figure S5 — KRAS/STK11 co-mutated tumors have an immune excluded phenotype via the xCell deconvolution algorithm [file crc-24-0153_figure_s5_supps5.pdf]

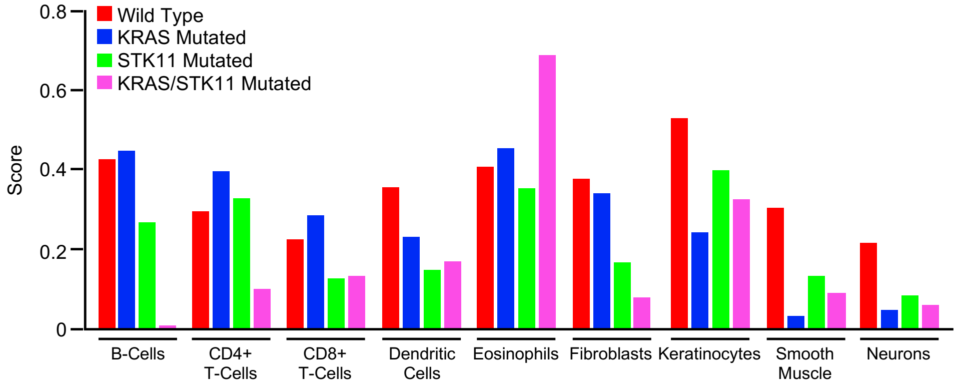

Figure S5. *KRAS*/*STK11* co-mutated tumors have an immune excluded phenotype via the xCell deconvolution algorithm
Immune and stromal cell types were estimated for patients using the xCell deconvolution algorithm and arranged by combined *KRAS* and *STK11* mutation status.
