# Supplementary material for: Loss of STK11 Suppresses Lipid Metabolism and Attenuates KRAS-Induced Immunogenicity in Patients with Non–Small Cell Lung Cancer
Source: Cancer Res Commun. 2024 Aug 30;4(8):2282–94. doi: 10.1158/2767-9764.CRC-24-0153 (PMC11362717; doi:10.1158/2767-9764.CRC-24-0153)
Supplement: Figure S6 — KRAS mutated tumors have an increased CD cytotoxicity score, which is lost with STK11 co-mutation [file crc-24-0153_figure_s6_supps6.pdf]

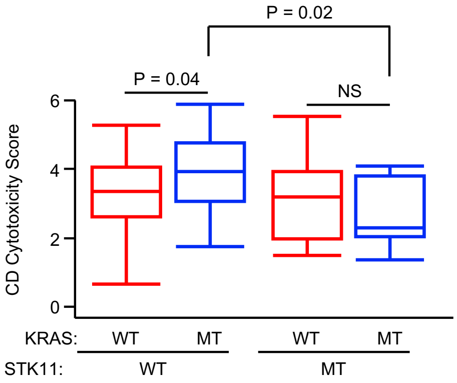

Figure S6. ***KRAS*** **mutated tumors have an increased CD cytotoxicity score, which is lost with** ***STK11*** **co-mutation**
CD cytotoxicity scores for patients arranged by combined *KRAS* and *STK11* mutation status.
