# Supplementary material for: Loss of STK11 Suppresses Lipid Metabolism and Attenuates KRAS-Induced Immunogenicity in Patients with Non–Small Cell Lung Cancer
Source: Cancer Res Commun. 2024 Aug 30;4(8):2282–94. doi: 10.1158/2767-9764.CRC-24-0153 (PMC11362717; doi:10.1158/2767-9764.CRC-24-0153)
Supplement: Figure S7 — F. Correlation between genes involved in lipid metabolism and those involved in immune processes [file crc-24-0153_figure_s7_supps7.pdf]

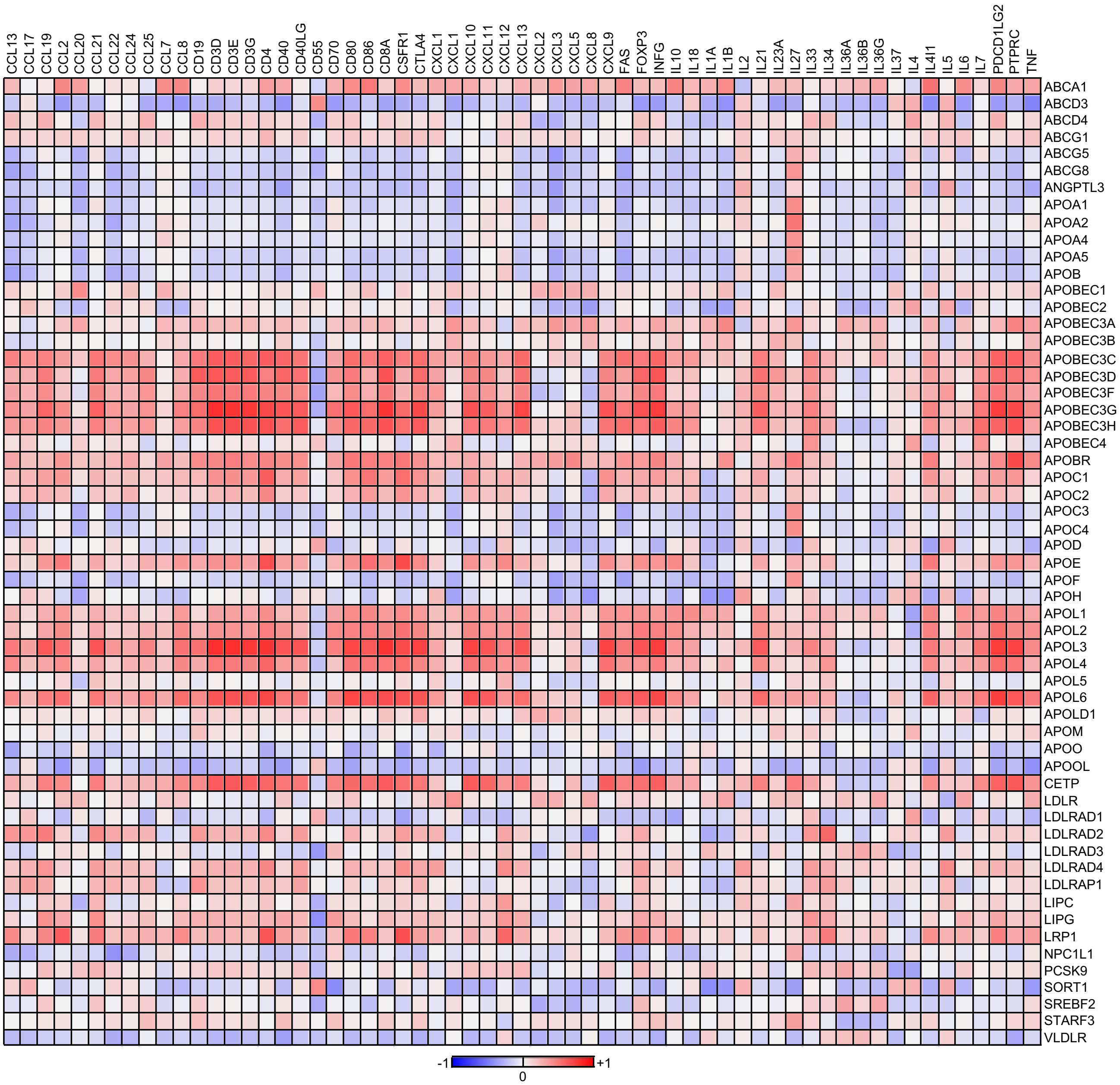

Figure S7. Correlation between genes involved in lipid metabolism and those involved in immune processes

Heatmap showing the Spearman's correlation coefficient for select genes involved in either lipid metabolism or immune cell processes.
