# Supplementary material for: Loss of STK11 Suppresses Lipid Metabolism and Attenuates KRAS-Induced Immunogenicity in Patients with Non–Small Cell Lung Cancer
Source: Cancer Res Commun. 2024 Aug 30;4(8):2282–94. doi: 10.1158/2767-9764.CRC-24-0153 (PMC11362717; doi:10.1158/2767-9764.CRC-24-0153)
Supplement: Figure S8 — Tumor stage and PD-L1 expression independently associate with overall survival [file crc-24-0153_figure_s8_supps8.pdf]

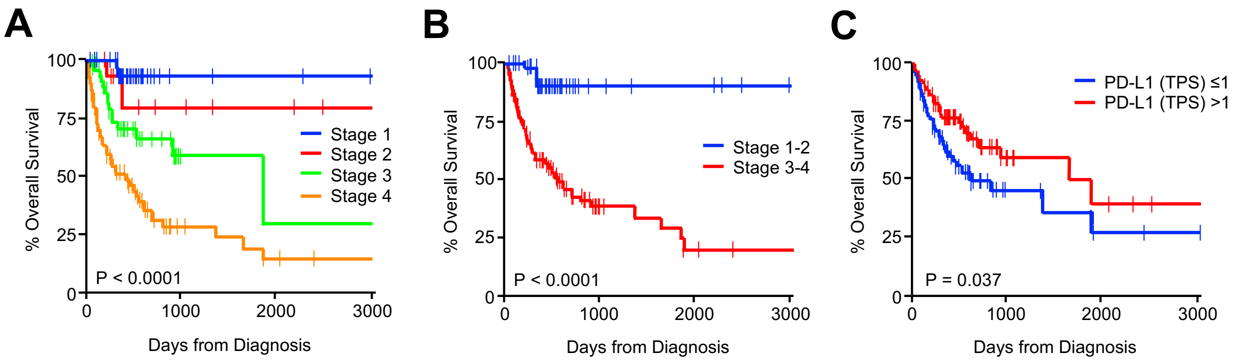

Figure S8. Tumor stage and PD-L1 expression independently associate with overall survival
Kaplan–Meier plots indicating months of overall survival for NSCLC patients arranged by **(A,B)** tumor stage or **(C)** PD-L1 TPS.
